# Colonic Delivery of Celastrol-Loaded Layer-by-Layer Liposomes with Pectin/Trimethylated Chitosan Coating to Enhance Its Anti-Ulcerative Colitis Effects

**DOI:** 10.3390/pharmaceutics13122005

**Published:** 2021-11-25

**Authors:** Jing Xian, Xuemei Zhong, Huan Gu, Xiao Wang, Jiaxin Li, Jingjing Li, Yihan Wu, Chen Zhang, Jinming Zhang

**Affiliations:** State Key Laboratory of Southwestern Chinese Medicine Resources, Pharmacy School, Chengdu University of Traditional Chinese Medicine, Chengdu 611130, China; xianjing4320@126.com (J.X.); zxm6781112@126.com (X.Z.); guhuan42@163.com (H.G.); wangxiao12643@126.com (X.W.); llijiiaxin@163.com (J.L.); ljj805812@gmail.com (J.L.); yihanwuone@126.com (Y.W.)

**Keywords:** trimethylated chitosan, layer-by-layer, celastrol, liposomes, ulcerative colitis

## Abstract

Herein, a flexible oral colon-targeting delivery system, mediated by electrostatic layer-by-layer alternate deposition with pectin-trimethyl chitosan (TMC) onto liposomes-loading celastrol (Cel/PT-LbL Lipo), was fabricated to enhance anti-UC efficacy. Along with layer-by-layer coating, Cel/Lipo exhibited surface charge reversal, a slight increase in particle size, and a sustained drug release profile in a simulative gastrointestinal tract medium. Based on its bilayer coating of polysaccharides, Cel/PT-LbL Lipo alleviated cytotoxicity of celastrol in colon epithelial NCM460 cells. Due to the strong mucoadhesion of TMC with mucin, PT-LbL Lipo benefited colon localization and prolonged retention ability of its payloads. Ultimately, Cel/PT-LbL Lipo significantly mitigated colitis symptoms and accelerated colitis repair in DSS-treated mice by regulating the levels of pro-inflammatory factors related to the TLR4/MyD88/NF-κB signaling pathway. Collectively, this study demonstrates that the pectin/trimethylated chitosan coating may allow for Cel/PT-LbL Lipo to function as a more beneficial therapeutic strategy for UC treatment.

## 1. Introduction

Ulcerative colitis (UC) is a chronic idiopathic inflammatory disease that continuously occurs from the distal to the proximal ends of the colon. Typical symptoms include bloody diarrhea, abdominal pain, fecal urgency, and tenesmus [1]. Over the last few decades, recommended anti-UC agents have included anti-inflammatory drugs like 5-aminosalicylic acid, steroids, and immunosuppressive agents [2]. However, owing to the complex etiology of UC, these agents have only limited therapeutic outcomes and are coupled with serious side-effects such as nephrotoxicity, neurotoxicity, and intestinal flora disturbance [3]. Given this, increasing attention has been paid to screening effective phytochemicals, as this is a promising, alternative strategy to finding novel agents [4]. Of those screened, natural products like curcumin, silymarin, and rhein have been shown to effectively ameliorate UC-related symptoms and help regulate levels of inflammatory molecules [5]. Celastrol (Cel) is a principal bioactive ingredient of *Tripterygium wilfordii* Hook. f. [6]. Importantly, it exhibits multiple promising biological activities for the treatment of many inflammation-related diseases, such as metabolic illnesses (e.g., atherosclerosis, obesity), autoimmune illnesses (e.g., arthritis, inflammatory bowel illnesses), central nervous system illnesses (e.g., Alzheimer’s disease, cerebral ischemia), and organ fibrosis (e.g., liver fibrosis, renal fibrosis) [7]. In particular, the anti-UC effects of Cel have been demonstrated across multiple measures, including modulating oxidative stress, inflammatory cytokines, autophagy, and intestinal homeostasis [8,9,10]. Finally, Cel has been shown to significantly ameliorate experimental colitis in either IL-10 deficient mice or dextran sodium sulphate-induced colitis mice. Despite these promising findings, its further clinical application has been largely impeded because of its low aqueous solubility (13.25 ± 0.83 μg·mL^−1^ at 37 °C) and poor stability in the gastrointestinal (GI) tract [11].

Currently, oral administration is the preferred route of UC treatment, owing to its convenience, cost-effectiveness, and elevated patient compliance [12]. Given this, several lipid- and polymer-based nanocarriers have been developed for the oral delivery of Cel. However, current research regarding oral delivery of Cel has been primarily concentrated on promoting its oral bioavailability. Rather than enhanced intestinal absorption, a few nano-vehicles have been designed to target distant, inflamed sites. It has been shown that nano-scaled drug delivery systems may be promising approaches for efficient colon delivery, with potentially effective outcomes in the treatment of UC [13]; however, the development of a nanocarrier with good GI stability and ideal colon tissue permeability after oral administration remains highly challenging. Moreover, considering *Tripterygium wilfordii* Hook. f. as a poisonous plant, the potential toxicity of Cel should not be overlooked. Although Zhang et al. reported that the oral absolute bioavailability of Cel in rats is only 17.06% [14], its intestinal absorption was greatly improved through dosage forms and nano-systems. However, these amounts of Cel absorbed in the small intestine would result in serious systemic toxicity. Therefore, minimizing the GI absorption and improving local targeting as well as tissue permeability in colon lesions is fundamental to maximizing the therapeutic efficacy at inflamed sites and reducing the side-effects of Cel.

Given that natural polysaccharides are effectively degraded by the physiological environment of the colon, considerable research has recently focused on developing polysaccharide-based micro/nanocarriers for colon delivery. Some polysaccharides, like chitosan [15], pectin, hyaluronic acid, and sodium alginate, have been used to coat the surface of drug delivery systems. This has resulted in enhanced GI stability and colon targeting [16,17,18,19,20]. A polyelectrolyte complex between chitosan (polycation) and pectin (polyanion) has drawn increased attention for its enteric-coated materials. Chitosan/pectin complexes exhibit high mucoadhesive properties and a pH-dependent swelling sensitivity, indicating they are compatible for colon-delivery [21]. For instance, Alkhader et al. demonstrated the mucoadhesive property of chitosan-pectin nanoparticles (NPs), which maintained their integrity in the upper gastrointestinal tract and mucoadhered in the colon lesion [22]. However, chitosan exhibited poor solubility in physiological media, but was soluble at the low pH of the stomach. This remains a limitation and would prevent widespread application.

Here, we have used the chitosan derivative, trimethyl chitosan (TMC)—which possesses good solubility across a broad pH range. Moreover, it has strong mucoadhesion and other advantages including the ability to transiently open tight junctions and interact with negatively charged mucin glycoprotein. When compared with chitosan, TMC allows for stronger adsorption on the NPs surfaces and overall enhanced intestinal permeability [23].

**Scheme 1:**
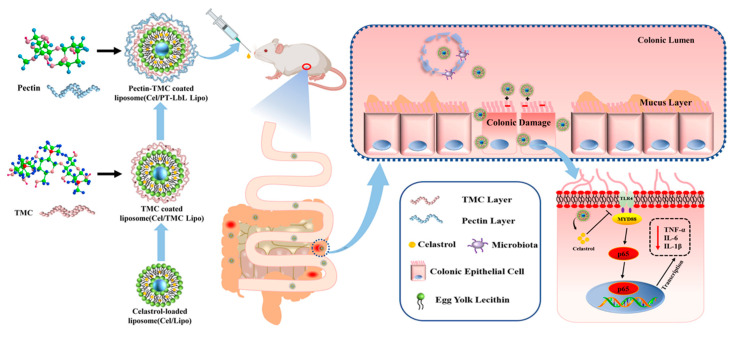
Schematic illustration of Cel/PT-LbL Lipo working against UC in mice. Oral administration delivery of pectin-TMC coated liposomes acts against UC by protecting the colonic epithelial cells from damage and anti-inflammation.

As a proof-of-concept demonstration, here we constructed pectin/TMC-functionalized layer-by-layer liposomes loaded with Cel (Cel/PT-LbL Lipo). This was accomplished using electrostatic alternate deposition on liposomes. We hypothesized that Cel/PT-LbL Lipo would exhibit an efficient anti-UC ability, owing to the following three factors (Scheme 1): (i) pectin-coated TMC multiple layers would provide flexible resistance in GI conditions; (ii) along with the hydrolysis of the pectin layer in the colon, the cationic TMC-coated Lipo exhibited high mucoadhesiveness, cellular uptake, and colonic tissue permeability; (iii) released Cel from the liposomes effectively regulated inflammation-related pathways. Collectively, the physicochemical properties, in vitro drug release, cell uptake, colon adhesion, and anti-UC effects of Cel/PT-LbL Lipo were investigated in this study. It is hoped that these results may be beneficial for the colon-targeted delivery of anti-UC agents.

## 2. Materials and Methods

All information regarding the experimental materials and methods is in the Supplementary Materials.

## 3. Results and Discussion

### 3.1. Synthesis and Characterization of TMC

**Figure 1:**
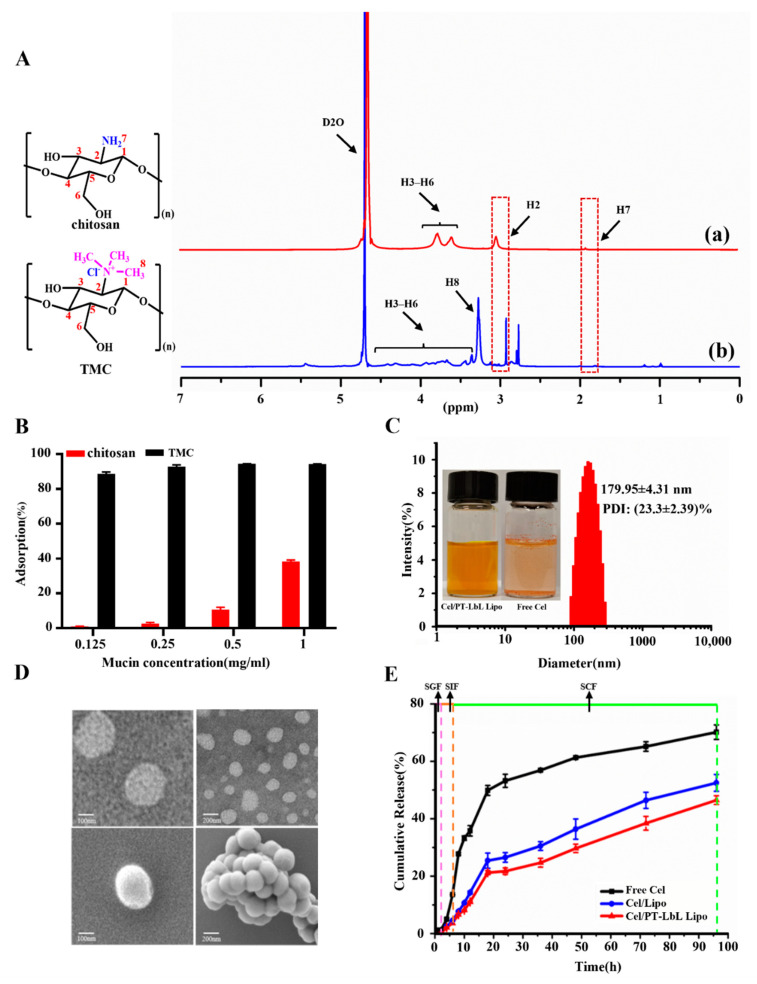
Characteristics of TMC polymer and Cel/PT-LbL Lipo. (**A**) ^1^H NMR spectra of chitosan (a) and TMC (b). (**B**) Mucoadhesive activity of chitosan and TMC with mucin. (**C**) Size distribution and appearance of Cel/PT-LbL Lipo suspension. (**D**) Representative TEM (upper row) and SEM (bottom row) images of Cel/PT-LbL Lipo. Scale bar = 200 nm. (**E**) In vitro release profiles of Cel from Free Cel, Cel/Lipo, and Cel/PT-LbL Lipo in three simulated GI tract mediums at 37 °C.

TMC polymers are synthesized using a methylation reaction of chitosan with methyl iodide. The introduction of trimethyl groups on the main amino groups of the repetitive monomers in chitosan was shown by ^1^H NMR spectra. As shown in Figure 1A, some representative signals derived from the saccharide unit of TMC were observed, including signals at the following: 2.16 ppm relating to the methyl hydrogen atoms of the acetamide group, as well as 3.15 ppm (H2), 4.69 ppm (H1), and 3.50~4.25 ppm (H3–H6) [24]. The new peaks at 2.87 and at 3.35 ppm (H8) indicated the incorporation of TMC (Figure 1A(b)). The N-trimethylated groups were labeled as ^+^NT sites. According to quaternization degree (DQ) calculation formula from the integral of H2 peak at 3.10 ppm and hydrogen atoms of ^+^NT peak at 3.35 ppm, the DQ of TMC was approximately 26%. Additionally, the chemical shift at 2.87 ppm also indicated the presence of N-dimethylated sites (-N(CH_3_)_2_). Some other characteristics used to compare TMC and chitosan include solubility condition, SEM, FTIR and XRD; data regarding these comparisons are shown in Supplementary Materials Figure S1. As shown, TMC had a similar crystal structure as chitosan, but with much better water solubility.

To evaluate whether the derivatization of chitosan into TMC influenced the positive charge, we compared the zeta potential of TMC and chitosan. As shown, the zeta potentials of solutions of TMC and chitosan were 7.36 ± 0.87 mV and 6.23 ± 1.11 mV, respectively. The positive potential of TMC means it has potential to be used to coat the surface of liposomes along with layer-by-layer coating of pectin; something that also enhanced the tissue penetration of NPs [25].

### 3.2. In Vitro Mucin Mucoadhesion Assay

Various studies have reported the molecular interaction amongst chitosan and mucin, mainly owing to electrostatic attractions, hydrogen bonding, and hydrophobic interactions [26,27]. The interaction of chitosan and its derivates with mucin has been commonly used to evaluate their mucoadhesive properties. As shown in Figure 1B, both 2 mg of chitosan and TMC at the polymer adsorbed mucin across a concentration range of 0.125~1 mg/mL. However, TMC exhibited much stronger adsorption effects than chitosan. Moreover, 88.5~94.3% of mucin was adsorbed by TMC, while less than 50% of mucin could be adsorbed by chitosan. To investigate the interaction mechanism of TMC with mucin, we used a molecular docking approach. As shown in Supplementary Materials Figure S1D, the docking score was represented by the Gibbs free energy of binding (ΔG), which was −7.59 kcal/mol. TMC binds to two glycine residues on Mucin 1 (MUC1). Collectively, these results show that TMC combined well with mucin.

### 3.3. Characterization of Cel/PT-LbL Lipo

Cel/PT-LbL Lipo were prepared based on a layer-by-layer coating on the surface of negatively charged liposomes, which were obtained using a thin film hydration method. As shown in Figure 1C and compared with the poor solubility of Cel, the Cel/PT-LbL Lipo formed a homogeneous suspension with opalescence. The drug encapsulation efficiency (EE) and loading efficiency (LE) of Cel/PT-LbL Lipo were (94.45 ± 2.71)% and (1.26 ± 0.38)%, respectively. The characteristics of Cel/Lipo, Cel/TMC Lipo, and Cel/PT-LbL Lipo are summarized in Table 1.

Given the charge adsorption between the anionic group (-PO_4_^2−^) in lecithin and the cationic group (-N(CH_3_)_3_^+^) of TMC polymer, the addition of TMC readily coated the liposomes. After successively coating with TMC and pectin, the particle size of Cel/PT-LbL Lipo had increased slightly from 166.68 nm to 179.95 nm, compared with Cel/Lipo and Cel/TMC Lipo. Liu et al. reported that the increased particle size of liposomes resulted from the increased film thickness of the polyelectrolyte on the surface of liposomes [28]. Moreover, the charge was notably reversed (in Supplementary Materials Figure S1E). The negative potential of Cel/Lipo at −5.7 ± 0.15 mV transferred into 3.3 ± 0.32 mV of Cel/TMC Lipo. Nevertheless, along with the pectin coating on TMC, the potential of Cel/PT-LbL Lipo dropped to −10.0 ± 0.15 mV. Transmission electron microscopy (TEM) and scanning electron microscopy (SEM) analyses (Figure 1D) indicated a roughly spherical morphology of Cel/PT-LbL Lipo with the relatively uniform size.

### 3.4. In Vitro Drug Release Studies in Different Simulated Gastrointestinal Media

In Vitro Cel release profiles from Cel/Lipo and Cel/PT-LbL Lipo in simulated gastric fluid (SGF) for 2 h, simulated intestinal fluid (SIF) for 4 h and simulated colonic fluid (SCF) for 90 h are shown in Figure 1E and indicate how gastrointestinal transmission would occur. Compared with Free Cel, both Cel/Lipo and Cel/PT-LbL Lipo exhibited sustained drug release profiles. During the first 2 h in SIF, 5.01%, 2.50% and 2.14% of Cel were released from Free Cel, Cel/Lipo, and Cel/PT-LbL Lipo, respectively. Likewise, a small amount of Cel was released from Cel/Lipo and Cel/PT-LbL Lipo in SIF, indicating that the TMC coating effectively eliminated any burst of drug release in either the stomach or small intestines. Mediated by enzymolysis by microorganism from colonic digesta, Cel/Lipo and Cel/PT-LbL Lipo exhibited much faster drug release profiles in comparison with either SIF or SGF. More specifically, more than 20% of Cel was released during the first 12 h in SCF. This was attributed to the flexibility of the natural polysaccharides, which were easily degraded by the colonic microbiota and allowed for maximal drug release at the inflamed area. However, due to the dual-coating of the polyelectrolytes, Cel/PT-LbL Lipo still exhibited slower drug release profiles than Cel/Lipo during the whole experimental period, which showed that the Cel/PT-LbL Lipo afforded regulated drug release in the GI tract and exhibited their latency for controlled drug release in the colon.

As demonstrated, pectin is an anionic polysaccharide, which cannot digest in an upper gastrointestinal tract and poorly soluble in acid pH condition. Therefore, the acidic pH value cannot destroy the pectin layer, as well as the polyelectrolyte complexes between TMC and pectin. The oral delivery advantages of polyelectrolyte complexes constructed with chitosan and pectin have been well demonstrated in previous studies [29,30,31]. We generated the polyelectrolyte complexes between TMC and pectin during the drug delivery system preparation at neutral pH value, by the layer-by-layer via electrostatic attraction. These polyelectrolyte complexes can keep stable in the stomach due to the hydrophobicity of the pectin layer, so that Cel/PT-LbL Lipo in SGF shows a low drug release rate. Nevertheless, pectin can be digested by pectinolytic enzymes produced by the colonic microflora and entirely absorbed in the colon, so Cel/PT-LbL Lipo in SIF exhibited a much higher drug loading rate. Thus, mediated by the pH-controlled release profile of polyelectrolyte complexes between TMC and pectin, Cel/PT-LbL Lipo could steadily exist in the stomach, before dissociating and releasing its payload in the colon environment.

### 3.5. In Vitro Intracellular Uptake Study

Given that the pectin layer of Cel/PT-LbL Lipo was degraded by the intestinal flora first, we investigate the role of TMC Lipo on the cellular uptake of payloads, the fluorescent dye coumarin 6 (C_6_) was loaded into TMC Lipo. Free C_6_ and C_6_-loaded liposomes without any surface coating acted as controls.

**Figure 2:**
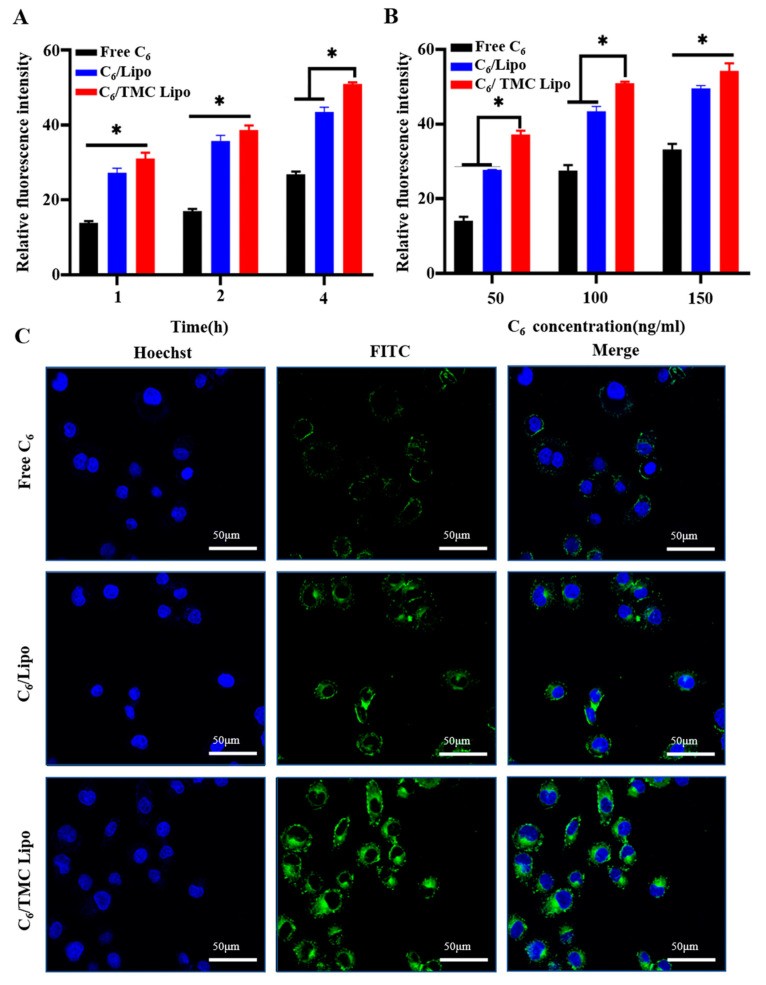
Cellular uptake advantages of C_6_/TMC Lipo on colonic epithelial NCM460 cells. (**A**) Quantitative intracellular accumulation of various C_6_ formulations with the equivalent C_6_ concentration of 100 ng/mL in NCM460 cells for 1, 2, and 4 h incubation, respectively. (**B**) Quantitative intracellular accumulation of various C_6_ formulations with a series of C_6_ concentration at 50 ng/mL, 100 ng/mL, and 150 ng/mL in NCM460 cells for 4 h, respectively. (**C**) Qualitative intracellular accumulation images of various C_6_ formulations with the equivalent C_6_ concentration of 100 ng/mL in NCM460 cells for 4 h incubation. ANOVA, Dunnett’s test; * *p* < 0.05.

First, we used flow cytometer (FCM) to quantitatively analyze the cellular uptake profiles of various C_6_ formulations after incubation with NCM460 cells for either different times or with different C_6_ amounts. As shown in Figure 2A, cellular uptake of all C_6_’s formulations occurred in a time-dependent manner. The intracellular amounts of C_6_ gradually increased with increasing incubation time. Even so, C_6_/TMC Lipo exhibited much stronger cellular uptake capacity than others. The enhanced uptake of C_6_/TMC Lipo was attributed to the positive charge of the TMC layer. Additionally, Figure 2B shows that more C_6_ was internalized into NCM460 cells for C_6_-loaded Lipo versus Free C_6_ at C_6_ concentration of 50~150 ng/mL. Similarly, based on the potential positive charge derived from TMC layer, C_6_/TMC Lipo also exhibited significantly enhanced cellular internalization, compared with either Free C_6_ or C_6_/Lipo. These consequences indicated that the Lipo-regulated cellular uptake of C_6_ was also both time- and dose-dependent.

Likewise, the enhanced uptake of TMC Lipo in NCM460 cells was qualitatively evaluated using confocal laser scanning microscopy (CLSM). After 4 h cellular incubation, the culture medium containing various C_6_ formulations with an equal C_6_ amount of 100 ng/mL were removed. Cell nuclei were stained using a Hoechst stain. As shown in Figure 2C, the green fluorescence represents C_6_ loaded into cytoplasm, while blue represents the nucleus. The images showed that Free C_6_ was not significantly taken up by the cells; comparatively, either Lipo without coating or TMC Lipo exhibited more internalized C_6_ in the cytoplasmic region. Meanwhile, C_6_/TMC Lipo showed the strongest fluorescence in cells, which was in line with the quantitative result in our FCM analysis. Collectively, these results demonstrate that although liposomes have been widely shown to improve the cellular uptake of payloads mediated by membrane fusion, coating with TMC also promoted cellular uptake in NCM460 cells.

### 3.6. In Vitro Anti-Inflammatory Effects of Cel/PT-LbL Lipo

In view of the ideal anti-UC effects exhibited by Cel and related to its anti-inflammatory effects, we next evaluated whether Cel loaded in liposomes could influence these anti-inflammatory effects. To do so, we used both a UC mice model induced by dextran sulfate sodium (DSS) and DSS to induce colonic epithelial cell injury and inflammation in vitro.

**Figure 3:**
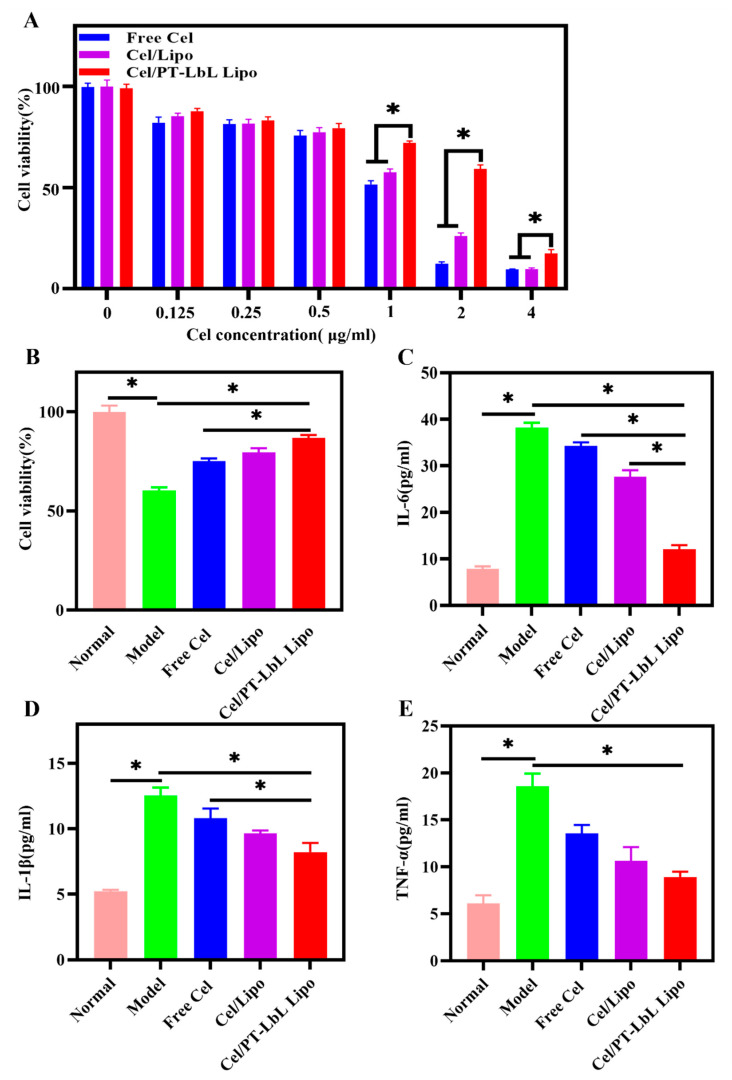
In vitro cytotoxicity and anti-inflammatory activity of Cel/PT-LbL Lipo on NCM460 cells induced by DSS. (**A**) Cell viability of NCM460 cells treated with various Cel formulations at a series of Cel concentrations (0–4 μg/mL) for 24 h. (**B**) Effects of various Cel formulations on cell viability of NCM460 cells induced by DSS. The expression levels of IL-6 (**C**), IL-1β (**D**), and TNF-α (**E**) in NCM460 cell culture medium, which were treated by various Cel formulations, meanwhile induced by 30 mg/mL DSS for 24 h. ANOVA, Dunnett’s test; * *p* < 0.05.

Due to the potential cytotoxicity of Cel, we first evaluated non-cytotoxic concentrations in NCM460 cells. As shown in Figure 3A, when the concentration of Cel was higher than 0.5 μg/mL, various Cel formulations exhibited—to certain degrees—cytotoxicity in NCM460 cells. Despite this, and mediated by multi-layer coating, Cel/PT-LbL Lipo notably alleviated the cytotoxicity induced by Cel when compared with Free Cel and Cel/Lipo. Therefore, to avoid the cytotoxicity induced by Cel formulations, we chose 0.5 μg/mL of Cel for all subsequent cellular experiments. Based on our preliminary experiment, 30 mg/mL of DSS was used to induce the cell injury model, which resulted in approximately 40% cell viability suppression and sharply ascending levels of inflammatory factors. As shown in Figure 3B, all Cel formulations alleviated the cell viability induced by DSS, in which Cel/PT-LbL Lipo exhibited the highest cell viability in NCM460 cells induced by DSS. This finding indicated that Cel/PT-LbL Lipo effectively alleviated the colonic epithelial injury. Furthermore, Figure 3C–E shows that Cel/PT-LbL Lipo reduced aberrant amounts of several pro-inflammatory cytokines induced by DSS, including IL-6, IL-1β, and TNF-α. Although Cel effectively lowered the levels of these pro-inflammatory cytokines in comparison with the Model group, Cel-loaded liposomes exhibited much higher capacity than Free Cel, which was in accordance with its prominent effect on alleviating cell viability suppression. These results demonstrate that Cel/PT-LbL Lipo significantly prevented cell injury and alleviated related inflammatory levels in vitro. The reasonable explanation for these results would be the enhanced cellular uptake of Cel/PT-LbL Lipo in NCM460 cells induced by the enhanced endocytosis effect of liposomes resulting from the TMC layer.

### 3.7. PT-LbL Lipo Preferentially Adheres to Colonic Mucosa Ex Vivo

Due to the buildup of positively charged proteins (e.g., bactericidal/permeability-increasing protein, transferrin, and antimicrobial peptides) on the damaged epithelial surface, previous work has shown the accumulation of positive charges on the inflammatory colonic mucosa [32]. Therefore, these negative-charged particles are thought to anchor on the surface of damaged colonic mucosa. To test this hypothesis, we next investigated the mucoadhesive effects of PT-LbL Lipo on colon mucosa ex vivo.

**Figure 4:**
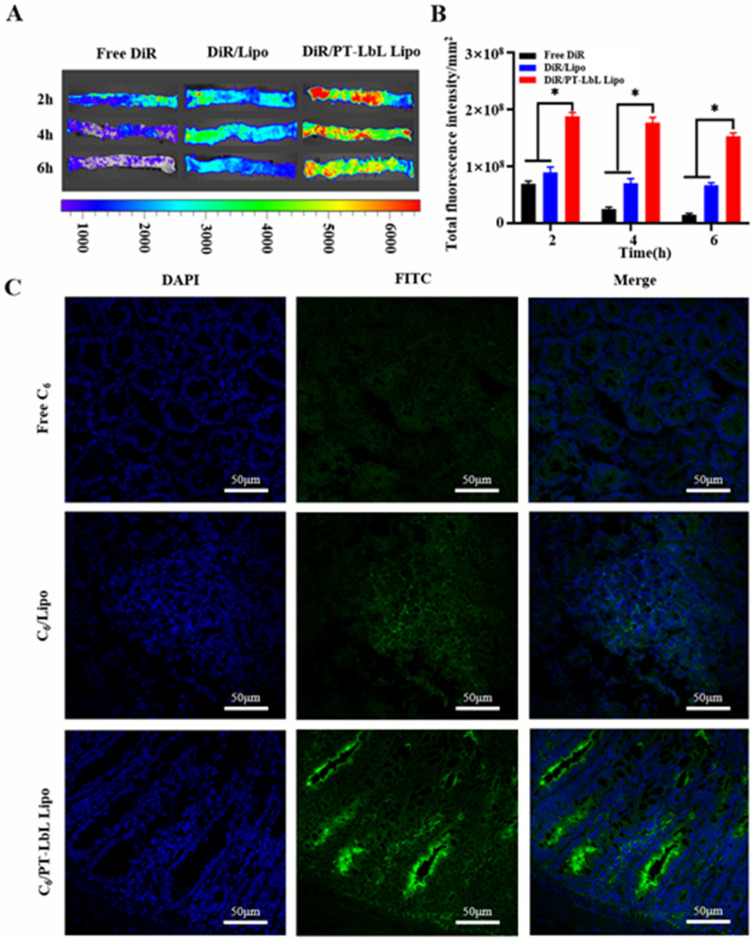
Ex vivo and in vivo colonic adhesion profiles of PT-LbL Lipo. (**A**) Residual fluorescence in UC rat colons ex vivo after incubation with various C_6_ formulations for 2, 4, and 6 h at 37 °C. (**B**) Quantification of fluorescence intensity in these colon tissues; Statistical significance was assessed (* *p* < 0.05). (**C**) After 12 h post-administration of various C_6_ formulations, UC mice were sacrificed and the colon tissues were collected. Fluorescence permeation in frozen sections were observed by CLSM. Green and blue represented the permeated C_6_ and cell nucleus stained by DAPI.

Mediated by the single-pass intestinal perfusion system, Free 1,1′-dioctadecyl-3,3,3′,3′-tetramethylindotricarbocyanine iodide (DiR) suspension, DiR/Lipo, and DiR/PT-LbL Lipo were syringed into the colon loop to mimic the enteric canal transmission in colon peristalsis. As shown in Figure 4A,B, the adhered fluorescence in the Free DiR group was weak and declined rapidly during 6 h of dynamic perfusion. Increased fluorescence was observed on the colon mucus after the administration of DiR/Lipo than Free DiR, with a slow decline in fluorescence. During the 6 h perfusion, the fluorescence intensity in the DiR/Lipo group was maintained. More importantly, the fluorescence intensity on colon mucus in the DiR/PT-LbL Lipo group was evidently much stronger than either the DiR/Lipo or Free DiR at each timepoint (* *p* < 0.05). Similarly, the fluorescence intensity in the DiR/PT-LbL Lipo group was not notably reduced, indicating that PT-LbL Lipo possessed good adhesion on inflammatory mucosa.

### 3.8. PT-LbL Lipo Facilitates Colon Tissue Penetration

Chitosan and its quaternized derivatives have been demonstrated as potent penetration enhancers across intestinal epithelia for poorly absorbable compounds [33]. Here, we assumed that the TMC layer on liposomes would benefit colonic mucous penetration. As shown in Figure 4C, few C_6_ were observed in the submucosa in the colon of mice administered Free C_6_. Faint green fluorescence was found in colon tissue after treatment of C_6_/Lipo, potentially because of the enhanced mucoadhesion of liposomes. However, the green fluorescence intensity in the C_6_/PT-LbL Lipo treatment group was much stronger than that of other groups, indicating that PT-LbL Lipo effectively accumulated and penetrated the colitis tissue after oral administration. These results suggest that PT-LbL Lipo facilitated the colonic penetration of payloads, resulting in increased therapeutic benefit to the submucosa of UC lesions.

### 3.9. Bio-Distribution of PT-LbL Lipo after Oral Administration in Mice

**Figure 5:**
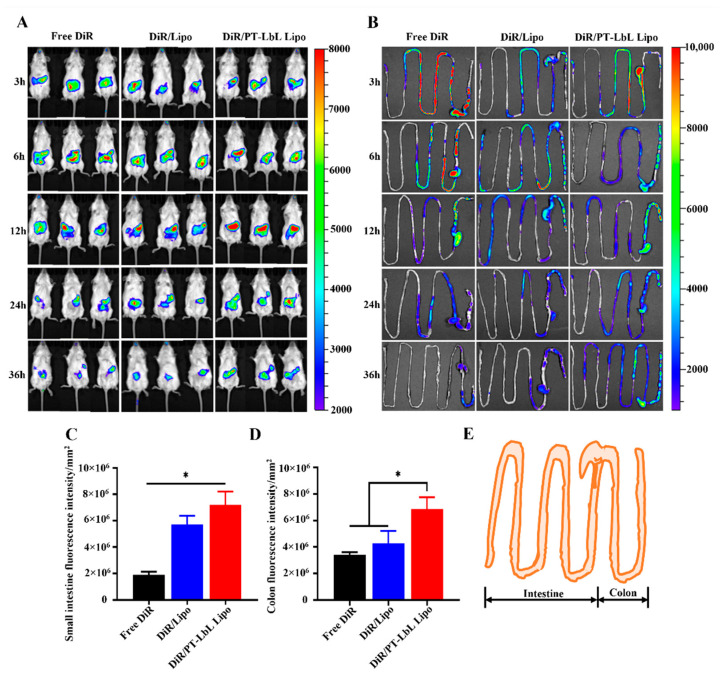
In vivo bio-distribution of PT-LbL Lipo. (**A**) After oral administration of Free DiR, DiR/Lipo, and DiR/PT-LbL Lipo at 3, 6, 12, 24 and 36 h, whole mouse body images were observed using a PerkinElmer IVIS Spectrum system. (**B**) Fluorescence retention images of small intestine and colon tissues following excision from UC mice at 36 h post-administration. (**C**) Fluorescence intensity quantification of small intestine across each preparation at the final endpoint. (**D**) Quantification of colon of ulcerative colitis mice across each preparation at the final endpoint. (**E**) Simulation diagram of the harvested intestine and colon tissue. ANOVA, Dunnett’s test; * *p* < 0.05.

To track the bio-distribution and colon targeting of PT-LbL Lipo along the mouse GI tract after oral administration in vivo, we used an optical living imaging experiment to monitor the location of fluorescently-labeled liposomes. The progression of DiR/PT-LbL Lipo along the mouse gastrointestinal tract was recorded by whole-body imaging over 36 h after oral administration (Figure 5A). During the experimental 36 h, the fluorescence in the GI tract exhibited rapid elimination in the Free DiR group, while the fluorescence elimination rate in the DiR/PT-LbL Lipo group was much slower, as it was mediated by the mucosal adhesion. Due to the curl of the small intestine and colon within the abdomen, it was hard to differentiate the disparate sections in the GI tract. Given this and to precisely estimate the colonic biodistribution, the whole mouse colon was removed at disparate time points and photographed for quantitative ex vivo analysis. As shown in Figure 5B, Free DiR suspension and DiR/Lipo without coating were eliminated more quickly from the mouse GI tract, compared with DiR/PT-LbL Lipo. At 36 h post-administration, the colonic fluorescence intensity in DiR/PT-LbL Lipo was much higher than that in either Free DiR or DiR/Lipo group. As shown in Figure 5C, we quantified the small intestine fluorescence at the last time point (36 h). The fluorescence intensity of DiR/Lipo, DiR/PT-LbL Lipo was stronger than Free DiR. Similarly, the quantitative results of 36 h colon are shown in Figure 5D. As indicated, the fluorescence of DiR/PT-LbL Lipo was significantly stronger than that of either Free DiR or DiR/Lipo (* *p* < 0.05). Taken together, the above data show that DiR/PT-LbL Lipo had better intestinal retention characteristics and colon localization ability.

### 3.10. In Vivo Therapeutic Effect of Cel/PT-LbL Lipo against UC

The DSS-induced UC mouse model is easy to generate and highly repetitive and is related to human UC [34]. Here, we investigated whether oral administration of Cel/PT-LbL Lipo would alleviate the clinical performance of DSS-induced UC in mice.

**Figure 6:**
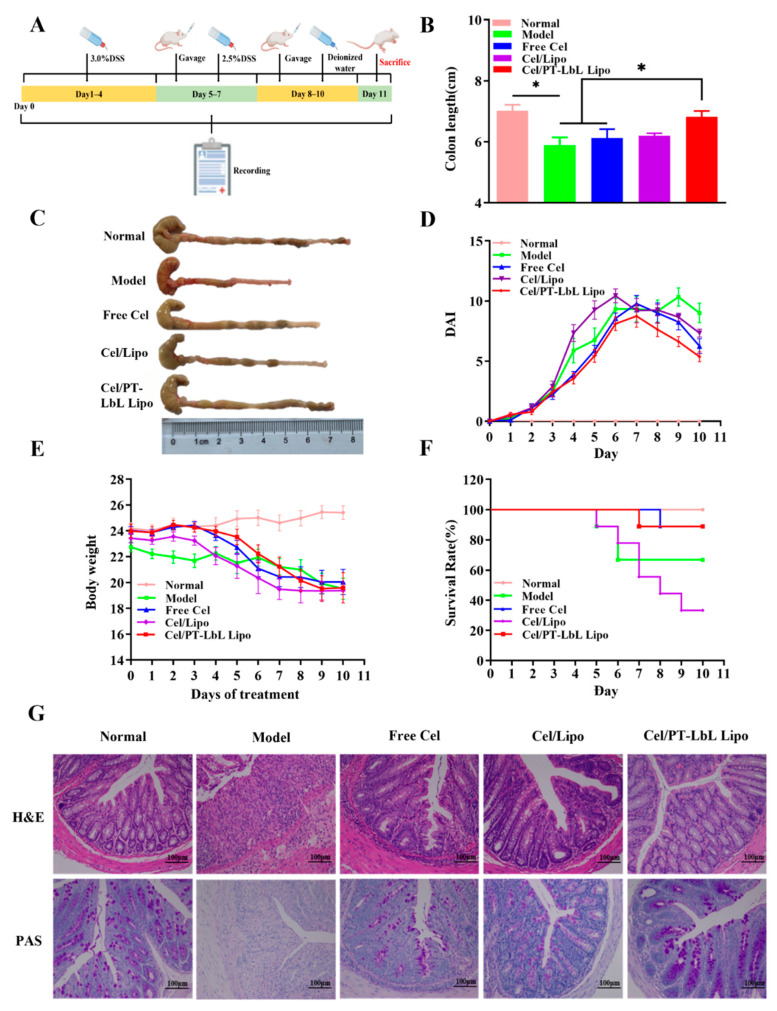
In vivo therapeutic outcomes of Cel/PT-LbL Lipo in DSS-induced UC mice model. (**A**) The graphical scheme of mice administration approach. UC mice model was induced by free drink of DSS solution (3.0%, *w*/*v*). At day 3, Free Cel (1 mg/kg), Cel/Lipo (0.5 mg/kg) and Cel/PT-LbL Lipo (0.5 mg/kg) were orally administered daily for a week. (**B**) Colon length; Statistical significance was assessed (* *p* < 0.05). (**C**) Representative colon photographs; (**D**) DAI scores; (**E**) Mice bodyweight curves during the whole experiment; (**F**) Survival rates; (**G**) H&E and PAS staining of colonic sections in different groups.

In the final experiment, the colons of mice in each group were collected and photographed, along with measurements of their respective lengths (Figure 6B). Gross observations (Figure 6C) of the excised colons showed that the liposomes group had obviously longer colon lengths compared with the Model group; moreover, that the Cel/PT-LbL Lipo treatment group had much longer colon lengths than those of the Cel/Lipo treatment group.

The severity of colonic inflammation was evaluated using a disease active index (DAI) score, which included the indexes of body weight decline, hematuria and stool consistency, as well as others [35,36]. As shown in Figure 6D, the DAI of the Model group was obviously bigger than that of the Normal group, whereas the Free Cel and Cel/PT-LbL Lipo groups had much lower DAI than the Model group. Additionally, body weight also made a significant contribution to estimating the efficacy of the agent in the DSS-induced UC mice model. Figure 6E shows the weight changes of each group over 10 days. On the third day—and except for Normal group—other groups began to lose weight. Collectively, these results clearly illustrate that Cel/PT-LbL Lipo was superior to other groups in the treatment of UC.

Next, we studied the therapeutic effect of each group on the survival rate. Except for the Normal group, all the other groups received DSS stimulation for 7 days, and different treatment methods were applied every day from the fourth day. Figure 6F shows the death of mice in each group within 11 days. The survival rates of the Model, Free Cel, Cel/Lipo and Cel/PT-LbL Lipo groups were 75.0%, 88.9%, 33.3% and 88.9% respectively. The above data showed that the survival rate of Cel/Lipo was lower—even lower than Free Cel—which suggested that liposomes may play a more toxic role because they are easily absorbed in small intestine. However, mediated by the colon-targeting capacity of PT-LbL shell, Cel/PT-LbL Lipo could avoid being absorbed in the intestine. Therefore, the survival rate of the Cel/PT-LbL Lipo group was significantly enhanced, compared with the Cel/Lipo group.

To observe the anti-UC effect in colon tissues collected from various groups, hematoxylin and eosin (H&E) staining and periodic acid-Schiff (PAS) staining were used to examine any histological changes. As shown in Figure 6G, compared with Normal group, Model group with DSS-induced UC resulted in severe mucosal injury, containing focal influx of inflammatory cells, colonic tissue necrosis and crypt damage. The crypt of the colon in the Cel/Lipo and Cel/PT-LbL Lipo groups were more complete than those of the DSS group, which indicated that both Cel/Lipo and Cel/PT-LbL Lipo ameliorated the signs and symptoms of colitis. PAS staining of colon tissue was also performed. These results showed that there were few blue and red positive spots in the cytoplasm of the Model group when compared with the Normal group, which indicated the mucus of the Model group was reduced and the colon was seriously damaged. The positive points of Cel/PT-LbL Lipo treatment group were as many as those of the Normal group, indicating that they repaired the colon mucosa and reduced the colon injury.

**Figure 7:**
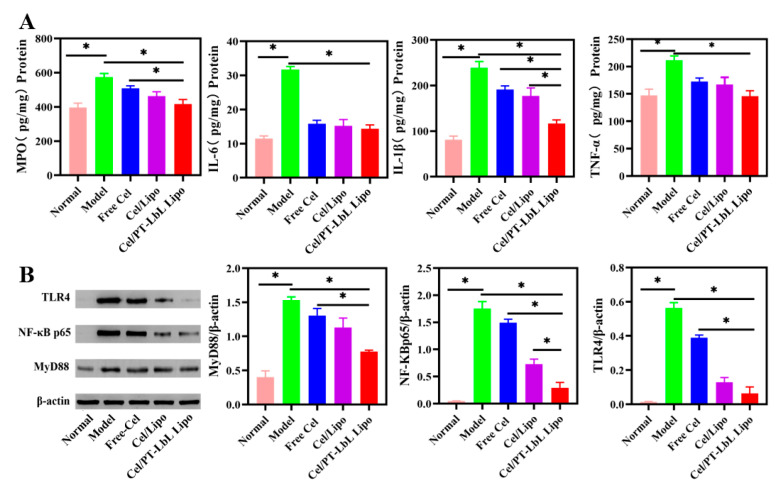
Colonic inflammatory cytokines and the expression of related protein in TLR4/NF-κB pathways. (**A**) Expression of MPO, IL-6, IL-1β, and TNF-α in murine colonic tissues by ELISA analysis. (**B**) The expression of TLR4, NF-κB p65, and MyD88 by Western blot analysis. Statistical significance was assessed (* *p* < 0.05).

It has been reported that inflammation severity is correlated with the amounts of pro-inflammatory cytokines [37]. As shown in Figure 7A, the secretion levels of major pro-inflammatory cytokines (e.g., MPO, IL-6, IL-1β, TNF-α) in the Model group were obviously higher than those in the Normal group. However, treatment with Cel/PT-LbL Lipo effectively reduced the levels of pro-inflammatory cytokines, while other treatment groups showed a slight decrease.

The TLR4/MyD88/NF-κB signaling pathway regulates the inflammatory response and plays an important role in the pathogenesis of UC [38]. Based on the DSS-induced NF-κB activation associated with TLR4 [39], we next sought to evaluate protein level by Western blotting. This was done to study the role of celastrol in this pathway, and to further study the liposomes. As shown in Figure 7B, and when compared with the Normal group, the total protein expression of TLR4, MyD88, and NF-κB p65 in the colon tissue of the Model group was significantly increased (* *p* < 0.05). However, the expression of these three proteins in Cel/PT-LbL Lipo group was significantly lower than any of the other groups (* *p* < 0.05). In short, Free Cel, Cel/Lipo, and Cel/PT-LbL Lipo all inhibited TLR4/NF-κB signaling pathway. Therefore, Cel/PT-LbL Lipo could facilitate the anti-UC effect, which would result from three aspects: (1) the TMC and pectin complex layer could benefit the colon-targeting and tissue adhesion of Cel/Lipo; (2) TMC and pectin complex layer could enhance the mucosal barrier; (3) the high amount of Cel/PT-LbL Lipo in colon could exhibit the suppression on tissue inflammation and related protein expression.

## 4. Conclusions

In the present study, we developed a flexible, oral, colon-targeting delivery system mediated by electrostatic layer-by-layer alternate deposition with pectin and trimethyl chitosan (TMC) onto liposomes for the treatment of ulcerative colitis. Consistent with our hypothesis, the fabricated Cel/PT-LbL Lipo had ideal resistibility to GI conditions, and rapid drug release in response to colonic pH value. Due to the strong mucoadhesion of TMC with mucin, PT-LbL Lipo allowed for better colon localization, with prolonged colonic retention and cellular uptake in enterocytes. Whether in vivo or in vitro and when compared with Cel/Lipo and Cel/TMC Lipo, Cel/PT-LbL Lipo had better pharmacodynamic effect, which were related to the TLR4/MyD88/NF-κB signaling pathway. In addition, and when compared with Free Cel and Cel/Lipo, Cel/PT-LbL Lipo had improved cytotoxicity and visceral toxicity. As expected, these results confirm our original hypothesis and demonstrate that Cel/PT-LbL Lipo not only increased anti-UC efficacy, but also reduced the side effects of Cel. These results prove that celastrol is an effective anti-UC drug, and provides a new idea and method for developing a drug delivery system based on celastrol.

## Figures and Tables

**Table 1:** Characterization of liposomal systems loaded with celastrol.

Liposomal System	Particle Size (nm)	Zeta Potential (mV)	Polydispersity Index (PDI, %)
Cel/Lipo	166.68 ± 3.00	−5.7 ± 0.15	20.23 ± 0.70
Cel/TMC Lipo	172.57 ± 2.87	3.3 ± 0.32	20.87 ± 3.07
Cel/PT-LbL Lipo	179.95 ± 4.31	−10.0 ± 0.15	23.33 ± 0.59

## Supplementary Materials

The following are available online at https://www.mdpi.com/article/10.3390/pharmaceutics13122005/s1, Figure S1: Characterization TMC and Cel/PT-LbL Lipo, Table S1: Particle size, zeta potential, and PDI of Cel/PT-LbL Lipo at day 0 and day 30 after storage at 4 °C for 1 month, Figure S2: Comparison of particle size, potential and TEM morphology of Cel/PT-LbL Lipo at day 0 (A, B, C) and day 30 (D, E, F) after storage at 4 °C for 1 month, Figure S3: Cellular uptake advantages of C_6_/TMC Lipo on colonic epithelial NCM460 cells, Figure S4: The representative H&E staining of heart, liver, spleen, lung, and kidney tissues.
