# Supplementary material for: Colonic Delivery of Celastrol-Loaded Layer-by-Layer Liposomes with Pectin/Trimethylated Chitosan Coating to Enhance Its Anti-Ulcerative Colitis Effects
Source: Pharmaceutics. 2021 Nov 25;13(12):2005. doi: 10.3390/pharmaceutics13122005 (PMC8703354; doi:10.3390/pharmaceutics13122005)
Supplement: Supplementary file 1 [file pharmaceutics-13-02005-s001.zip › pharmaceutics-1446243-supplementary.pdf]

pharmaceutics

Supplementary Materials: Colonic Delivery of Celastrol-Loaded Layer-by-Layer Liposomes with Pectin/Trimethylated Chitosan Coating to Enhance its Anti-Ulcerative Colitis Effects

Jing Xian, Xuemei Zhong, Huan Gu, Xiao Wang, Jiaxin Li, Jingjing Li, Yihan Wu, Chen Zhang and Jinming Zhang

1. Materials and Methods

1.1. Materials and Animal

Cel (CAS 34157–83–0, purity ≥ 98%) was purchased from Lemeitian Co., Ltd (Hangzhou, China). Chitosan (CAS 9012–76–4, 95% deacetylation grade) was purchased from Aladdin (Shanghai, China). Pectin (CAS 900-69-5, 74.0% esterification rate) was purchased from Sigma-Aldrich (Darmstadt, Germany). Dextran sulfate sodium (DSS, 36–50 kDa) was purchased from MP Biomedicals （Shanghai）Co., Ltd. (Shanghai, China). (1,1'-dioctadecyl-3,3,3',3'-tetramethylindotricarbocyanine iodide (DiR iodide) were purchased from Meilunbio® (Dalian Meilun Biotechnology Co., Ltd (Dalian, China)). Kits such as myeloperoxidase (MPO), tumor necrosis factor-alpha (TNF-α), interleukin-1β (IL-1β), and interleukin-6 (IL-6) were purchased from MultiScience (Lianke Biotech Co, Ltd., Hangzhou, China). Antibodies involved in the Western blot experiment were purchased from Abcam (Abcam Plc, Cambridge, UK).

Animal experiments were carried out in line with the instructions for the care and use of experimental animal issued by the Ministry of Science and Technology of China. Male Balb/c mice (22–25 g) were gained from SPF Biotechnology Co., Ltd. (Beijing, China). The experiments were carried out after approval by the Ethics Committee of Chengdu university of Traditional Chinese Medicine (permit no. CDU2019S183). The mice were reared under normative environment and provided with fodder and drinking water. In all animal experiments, the welfare of the animals was guaranteed.

1.2. Synthesis and Characterization of TMC

Based on the previous report by Chandrakantsing V et al [1]. TMC was synthesized by reductive methylation reaction of methyl iodide (CH_3I, a methylating agent) and chitosan in the presence of sodium hydroxide (NaOH) at 60 °C. Simply, 2.0 g of chitosan and 4.8 g of sodium iodide (NaI, catalyzer) were dissolved in 80.0 mL of a base N-methyl-2-pyrrolidinone (NMP) at three-neck flask, then placed on a thermostatic oil bath at 60 °C, and stirred continuously until chitosan was completely dissolved. Subsequently, 11.0 mL of NaOH solution was added (15%, *w/v*) and 11.5 mL of methyl iodide before magnetic stirring. The obtained solution was precipitated with 200 mL ethanol, centrifuged, washed with acetone, and dried. After dissolving in 80.0 mL NMP at 60 °C, 4.8 g of NaI, 11.0 mL of NaOH solution and 7.0 mL CH_3I were added. The mixture was stirred at 60 °C for 30 min in advance, and then mixed with 2.0 mL of CH_3I and 0.6 g of NaOH for 1 h reaction. The obtained N,N,N-trimethyl chitosan iodide (TMI) was precipitated with 200 mL ethanol, centrifuged, washed with acetone twice and dried in a vacuum. The TMI was dissolved in 40.0 mL of 10% NaCl solution to exchange chloride for iodide ion. After being precipitated with ethanol and washed by ethanol and ether alternatively, the TMC product— a gray-white water-soluble powder—was obtained after freeze-drying. The quaternization degree (DQ) of TMC was calculated from deconvolution of the signals in the ^{1}H NMR spectra, mediated by the following Equation [2]:

$$DQ\% = \left[\frac{[(CH_3)_3]}{[H_2]}\frac{1}{9}\right] \times 100 \quad (1)$$

The ^1H NMR spectra of chitosan and TMC were tested by using an AVANCE NEO-600MHZ spectrometer (Bruker, Switzerland) with 1% hydrochloric acid D_2O and D_2O as solvents, respectively. Tetramethylsilane was employed as an internal standard. The superficial structure of chitosan and TMC were determined using scanning electron microscope and the infrared spectra of chitosan and TMC were recorded using a FTIR spectrophotometer (IR Tracer-100, Shimadzu, Tokyo, Japan). In addition, their X-ray diffraction (XRD) spectra were measured (D8 Advance, BRUKER, Karlsruhe, Germany) by scanning from 5 to 90°.

1.3. In Vitro Mucoadhesion Assay of TMC

The mucoadhesive properties of chitosan and TMC were compared by their bio-absorption with mucin [3]. Briefly, 2 mL of gradient mucin standard solutions were prepared to obtain standard calibration curves. Then, 0.2 mL of periodic acid reagent was added to each of them. The specimens were incubated in a water bath at 37 °C for 2 h. After that, 0.2 mL of Schiff reagent was added, and the absorbance of the solution was determined at 555 nm after 30 min with a microplate reader (SpectraMax® iD3, Molecular Devices, San Francisco, CA, USA). Likewise, chitosan and TMC were determined by the same procedure, 10 mg of sample of each was dispersed in the mucin solution, shaken, and centrifuged for 5 min, and the supernatant was used to measure the estimated free mucin content according to the standard curve calibration.

The interaction of TMC with mucin was also verified to investigate its mediation by molecular docking. Mucin 1 (MUC1), a cell surface mucin mostly expressed in mucosal tissues, has been demonstrated to increase in colitis tissue and play a role as a target for small molecule inhibitors [4]. The crystal structure of MUC1 (PDB ID:6TM6) was gained from the Protein Data Bank (http://www.rcsb.org, September 21, 2020), and the 3D structure of the TMC ligand came from chitosan (CID:129662530) that was downloaded from the PubChem database (https://pubchem.ncbi.nlm.nih.gov/PubChem, September 22, 2020). The structure was energy minimized with fixed heavy-atom coordinates using the Tripos force field for 10,000 iterations subject to a termination gradient of 0.05 kcal/ (mol, Å). The AUTODOCK software was used to match the molecular binding between TMC and MUC1.

1.4. Preparation of Cel/PT-LbL Lipo

Cel-loaded core liposomes were primarily made by regular thin-film hydration method [5]. Specifically, 2.0 mg Cel and the mixture of egg yolk lecithin and cholesterol at a mass ratio of 5:1 were dissolved in 15 mL ethanol. After vortexing for 1 min, the mixture was evaporated under reduced pressure for 1 h to form a dried thin film. The lipidic film was rehydrated with 8 mL PBS buffer at 40 °C under the assistance of vortex and ultrasound. The liposome suspension was sonicated for 5 min at 300 W in an ice bath using an ultrasonic cell disruption system, and then passed through a 0.22 μm millipore filter. The dispersed liposome suspension was stored at 4 °C for further experimentation.

The stock solution of TMC and pectin were prepared at concentrations of 0.5 mg/mL and 1.0 mg/mL, respectively. Double-layered liposomes were prepared through successive deposition of polysaccharides onto the surface of the core liposomes through layer-by-layer self-assembly methods via electrostatic powers [6]. Dropwise addition of 0.5 mL TMC solution into the core liposome solution was implemented with continuous stirring for 30 min. The Cel/TMC Lipo with single-layer coating was collected by a 0.22 μm millipore filter. After that, 1 mL of 1.0 mg/mL pectin solution was deposited onto TMC-coated liposomes by an identical method. The negatively charged pectin are autonomously coated onto the cationic surface of the TMC-coated liposomes by electrostatic adsorptions. The multiple layer-coated liposomes (Cel/PT-LbL Lipo) were stored at 4 °C until analysis.

1.5. Physicochemical Characterization of Cel/PT-LbL Lipo

The average hydrodynamic particle size, polydispersity index (PDI), and zeta potential of liposomes were determined by dynamic light scattering (DLS) using a laser particle

analyzer LITESIZER 500 (Anton Paar, Ligraz, Austria, Anto Paar Kalliope). The parameters regarding medium, refractive index, viscosity, and dielectric constant were water, 1.3303, 0.8903 mPa.s, 78.37 respectively. Average values were gained from three separate batches. The morphology of Cel/PT-LbL Lipo was observed by transmission electron microscopy (TEM, HT7800, Hitachi, Chiyoda, Japan), in which the copper mesh of the carbon plated support film was placed on the sealing film, the sample dropped to it. After ten minutes the excess solution from the edge part was removed after with pointed filter paper, before the dried support film was placed on the sealing film. The phosphotungstic acid dye was dropped on the sample, which was left to dye for 90 s. Following this the excess dye was removed with the filter paper, the sample was then clamped to the filter paper and observed after drying for 3 h. Morphology was also observed via scanning electron microscope (SEM, Zeiss Merlin Compact, Oberkochen, Germany). Here, the sample was obtained with a pipette gun, dropped onto the ultrasonic suspension on the silicon wafer or tin paper. This was then dried naturally or with an infrared lamp, before the silicon wafer or tin paper was placed on the sample table with electric conductive adhesive for testing.

1.6. Encapsulation Efficiency and Loading Efficiency

The encapsulated amounts of Cel in liposomes were determined using high-performance liquid chromatography equipped with photodiode array detector (HPLC-PDA) (LC 2030C 3D, SHIMADZU, Tokyo, Japan). The HPLC analysis was implemented with a C_{18} column (250 × 4.6 mm, 5 μm) at a maximum absorbance of 425 nm. The mobile phase was acetonitrile and 0.1% phosphoric acid solution (80/20, *v/v*). The flow rate was 1 mL/min. The column temperature was maintained at 25 °C while 10 μL of samples were injected. The linear range of Cel is 7.81~250.00 μg/ml and the equation of standard cure is Y=19647X−55113(R^2 = 0.9996). The LOQ and LOD were 485.00 ng/mL and 161.66 ng/mL, respectively.

To determine the drug loading in liposomes, 1 mL of Cel/PT-LbL Lipo suspension was added into a volumetric flask (5 mL), and then methanol was filled to volume. The liposome structure was destroyed by ultrasonic treatment for 2 min. The methanol suspension was filtrated using a 0.22 μm millipore filter before HPLC analysis. Additionally, the weight of Cel/PT-LbL Lipo was determined by a weight loss method. Specifically, 10 mL of Cel/PT-LbL Lipo was measured into a bottle pre-dried to a constant weight, then the 10 ml of Cel/PT-LbL Lipo sample was lyophilized, and the whole bottle was dried to a constant weight. The drug encapsulation efficiency (EE) and loading efficiency (LE) were defined as follows according to an anteriorly published method [7]:

$$\text{EE (\%)} = \text{Quantity of Cel loaded/ Quantity of Cel added} \times 100\% \quad (2)$$

$$\text{LE (\%)} = \text{Quantity of Cel loaded/Total quantity of liposomes harvested} \times 100\% \quad (3)$$

1.7. In Vitro Celastrol Release Study

In vitro Cel release profiles of Cel from Free Cel, Cel/Lipo, and Cel/PT-LbL Lipo were carried out using a dialysis method. Three types of drug release media, i.e., the simulated gastric fluid (SGF, pH 2.0), simulated intestinal fluid (SIF, pH 6.8), and simulated colonic fluid (SCF, pH 7.4) were applied to simulate the digestive tract environment. Here, 16.4 mL of dilute hydrochloric acid and 10.0 g pepsin was dissolved in about 800 mL water, before further water was added up to a total volume of 1000 mL, then shaken uniformly to prepare SGF. For SIF, 6.8 g of potassium dihydrogen phosphate was dissolved with 500 mL water before adjusting the pH to 6.8, 10.0 g of trypsin was also dissolved in a suitable amount of water, before the two solutions were mixed and further water was added to bring the volume to 1000 mL. For the SCF, this was prepared from the rat colon digesta according to the previous reference [8]. Briefly, the caecum was exteriorized from rat, its content suspended in double volumes of cold phosphate buffer saline (PBS) to give a final

caecal diluent. Then centrifuged at 3000 × g for 30 min for gaining a clarified supernatant including extracellular enzymes. Later, suitable PBS was added to obtain the dilute solution.

A suspension of 2 mL of Cel/Lipo, Cel/PT-LbL Lipo and Cel dissolved in DMSO were added into dialysis bags (5000 Da), respectively. The concentration of Cel was 270.26 μg/mL. The dialysis bags were sealed, and then soaked in tubes with 30 mL different releasing media. The tubes were stirred at 100 rpm at 37 °C. At predetermined intervals, 1 mL of release media was withdrawn for measurement and an equal volume of fresh releasing media were supplemented. The amount of Cel was then measured by HPLC with the above-described conditions. All the procedures were performed in triplicate.

1.8. Cell Culture

The human normal colonic epithelial cells NCM460 cell line was gained from Shanghai Gaining Biological Technology Co, Ltd. Cells were maintained in RPMI 1640 medium supplemented with 1%(*v/v*) penicillin and streptomycin (Servicebio, Wuhan, China) and 10% fetal bovine serum (FBS). The cell was grown at 37 °C under 5% CO_2.

1.9. Cellular Uptake In Vitro

The fluorescence probe coumarin 6 (C_6) was loaded to label the liposomes with the same approach as TMC Lipo. Cellular uptake profiles of liposomes were detected using a flow cytometer (FCM) (BD FACSVerse™, Franklin Lake, NJ, USA) and confocal laser scanning microscopy (CLSM, TCS SP8 SR; Leica, Weztlar, Germany).

NCM460 cells were cultured beforehand in 6-well plates at a density of 5 × 10^5 cells/well. After overnight incubation, Free C_6, C_6/Lipo, and C_6/TMC Lipo with an equal C_6 concentration of 100 ng/mL were added in wells for 1, 2, and 4 h incubation. The culture medium was discarded and the cells were washed with cold PBS. Additionally, to evaluate dose-dependency, a series of C_6 concentrations (at 50, 100, and 150 ng/mL) of Free C_6, C_6/Lipo, and C_6/TMC Lipo were added into NCM460 cells with 4 h incubation. Untreated NCM460 cells were used as control. Cells were gathered by trypsin and re-suspended in PBS for FCM assay. The fluorescence intensity was counted for 10,000 cells.

The cellular internalization of C_6/TMC Lipo was observed by CLSM. NCM460 cells were seed into 35 mm glass bottom culture dishes for 24 h. Then, the culture medium was substituted for fresh medium containing the equivalent C_6 concentration at 100 ng/mL of Free C_6, C_6/Lipo, or C_6/ TMC Lipo, and incubated for 4 h. Then, cells were washed with cold PBS followed by fixed with 4% paraformaldehyde for 10 min. The nuclei were stained with Hoechst 33342 for 10 min and then surveyed by CLSM.

1.10. Effects on Cell Proliferation of NCM460 Induced by DSS

Briefly, NCM460 cells were seeded in 96-well plates at a density of 6 × 10^3 cells/well and incubated overnight for cell adhesion. Cells were then incubated with Free Cel, Cel/Lipo, Cel/PT-LbL Lipo at a series of Cel concentrations (0–4 μg/mL) for 24 h. Cell viability was estimated by the CCK-8 assay (MultiSciences Lianke Biotech Co., Ltd, Hangzhou, China). Additionally, NCM460 cells were pretreated with DSS to simulate the colon mucosal injury of a UC lesion. Specifically, NCM460 cells were primarily exposed to 30 mg/mL DSS for 12 h and then the culture medium was changed by fresh medium including Free Cel, Cel/Lipo, or Cel/PT-LbL Lipo with the equivalent Cel concentration of 0.1 μg/mL Cel and 30 mg/mL DSS. For additional 24 h incubation, the cell viability was measured using CCK-8 assay.

1.11. Anti-Inflammatory Effects of Cel/PT-LbL Lipo In Vitro

The inflammatory cell model was produced and mediated by the injury to NCM460 cells by DSS. Specifically, NCM460 cells were plated in 24-well plates, and cell concentration was set as 1 × 10^5 cell/well. After overnight incubation, the culture medium was replaced by fresh medium containing Cel/PT-LbL Lipo with the concentration of Cel at 0.1 μg/mL) with or without 30 mg/mL DSS. After 24 h incubation, the cell supernatant was

gathered and some inflammatory factors containing IL-6, IL-1β, and TNF-α were measured by ELISA kits.

1.12. Ex Vivo Mucoadhesion and Penetration in Colon Tissue

To observe the ex vivo colonic tissue adhesion of liposomes, the near-infrared fluorescent probe DiR was encapsulated in liposomes and PT-LbL liposomes, named as DiR/Lipo and DiR/PT-LbL Lipo, respectively. Firstly, UC symptoms were induced in rats by successive oral gavage with 3% (*w*/*v*) DSS for 7 days. And then, the UC rats were sacrificed and the distal colon tissue excluding the anus was dissected. A colon loop of about 8 cm was captured and Free DiR, DiR/Lipo and DiR/PT-LbL Lipo suspension containing DiR concentration of 0.25 mg/kg were syringed into colon loop respectively. These perfusion suspensions were perfused into colon loop at a flow rate of 0.5 mL/min using an infusion pump (Qingpuhuxi, Shanghai, China). After single-pass intestinal perfusion for 2, 4, and 6 h at 37 °C, the colon loop was softly washed with 10 mL cold PBS (3×). The colon was opened longitudinally and ex vivo tissue images of the luminal side facing up were implemented immediately using an IVIS fluorescence imager.

Based on the tissue mucoadhesion test, we also further explored whether PT-LbL Lipo might benefit from penetration into the colon tissue after oral administration. Balb/c male mice (8 weeks of age, 22–24 g) were used in the animal experiments. Mice were group fed (25 °C), photoperiod (12:12-h light-dark cycle). All animals were adapted for 7 days before the experiment. Firstly, UC mice model was induced with 3% DSS treatment for continuous 7 days. And then, 0.2 mL of C_6 loaded liposomes (termed as C_6/Lipo, C_6/ PT-LbL Lipo, respectively) were administrated to UC mice by gavage at equivalent C_6 concentration (0.2 mg/kg). After 12 h post-administration, mice were sacrificed and the distal 3 cm of the colon was removed and quenched with liquid nitrogen. Then, the frozen tissues were sectioned and stained by DAPI for imaging with CLSM.

1.13. In Vivo Biodistribution of PT-LbL Lipo by Oral Administration

To observe the in vivo colonic accumulation of PT-LbL Lipo, UC mice were divided into three groups. Free DiR, DiR/Lipo and DiR/PT-LbL Lipo were each orally administered with a dosage of 0.25 mg/kg of DiR. Prior to imaging, animals were anaesthetized by isoflurane in oxygen, and placed in an imaging cradle. After the different time-points (3, 6, 12, 24, and 36 h), mice were photographed using a living imaging system (Caliper Life Sciences LIVIS Lumina Series PerkinElmer, Waltham, MA, USA). At the endpoint, mice were sacrificed, and the gastrointestinal tract was removed and imaged. The mean fluorescence intensity was quantified using PerkinElmer Living Image software in a standard-size ROI drawn around individual colon pieces.

1.14. In Vivo Therapeutic Evaluation

Mice were stochastically divided into 5 groups (6 mice per group): namely, (i) control (saline + no DSS treatment), (ii) UC model (saline + DSS treatment), (iii) Free Cel (CMC Na suspension containing Cel + DSS treatment), (iv) Cel/Lipo (Cel/Lipo suspension + DSS treatment), (v) Cel/PT-LbL Lipo (Cel/PT-LbL Lipo suspension + DSS treatment). Particularly, both Cel/Lipo and Cel/PT-LbL Lipo were orally administered with the equivalently Cel dose of 0.5 mg/kg daily, while Free Cel was administered at a dose of 1 mg/kg. Mice body weights were recorded every day during the whole experiment. Before executing the animals, colitis was quantified with disease activity index (DAI) by assessing weight decline (0–4), fecal bleeding (0–4), and stool consistency condition (0–4), as previously described [9]. The concentrations of pro-inflammatory cytokines MPO, IL-6, IL-1β, TNF-α in colon tissues were detected with ELISA kits. As to histological examination, organs (heart, liver, spleen, lung, and kidney) and colon were fixed in 4% formalin solution and imbedded in paraffin, sectioned into slices (5 μm) and stained by H&E. Western blotting was carried out to determine the expression level of MyD88, NF-κB p65, and TLR4. Colon tissue samples were obtained and homogenized using a radio immunoprecipitation assay lysis buffer. Main antibodies containing anti-NF-κB p65 (1:1000), anti-MyD88 (1:1000),

anti-TLR4 (1:1000), and anti-β-actin (1:3000) antibodies were used as control. Meanwhile, the survival rates for various groups were recorded during the whole experimental period.

1.15. Statistical Analysis

All the data were presented as mean ± standard error of the mean (SEM) from at least three replications. One-way ANOVA followed by Duncan's multiple range test (* $p < 0.05$) was applied for statistical analysis. $p < 0.05$ was considered statistically significant.

2. Results

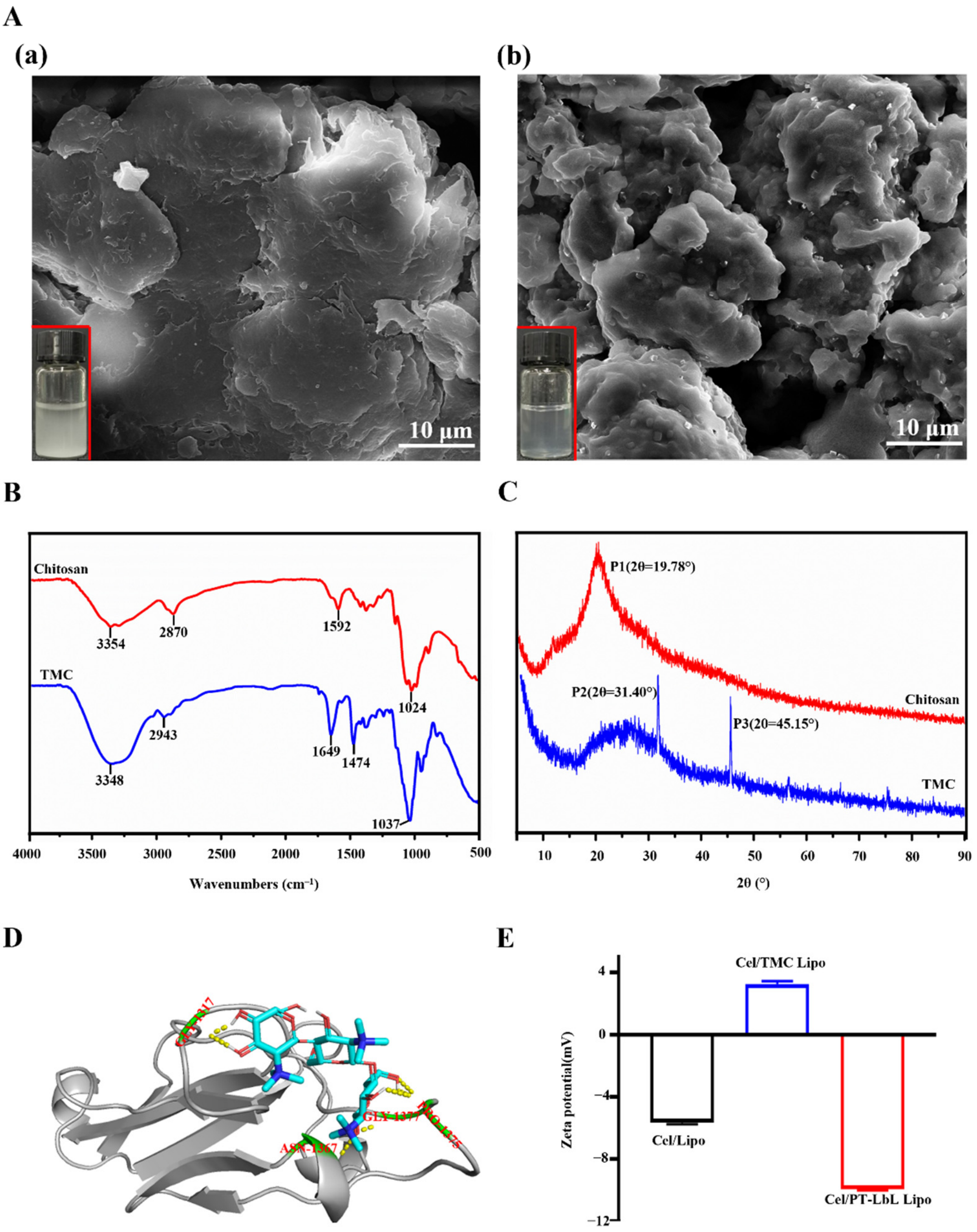

Figure S1. Characterization TMC and Cel/PT-LbL Lipo. (**A**) The SEM images and solubility of chitosan (a) and TMC (b). (**B**) FTIR spectra of chitosan and TMC. (**C**) XRD diffractograms of chitosan and TMC. (**D**) Molecular docking model of the interaction between TMC and mucin. (**E**) Zeta potential changes of Cel/Lipo, Cel/TMC Lipo, and Cel/PT-LbL Lipo.

As shown in Figure.S1A, the superficial structures of chitosan (a) and TMC (b) were determined using scanning electron microscope. The surface of chitosan is rough and the texture is clear while TMC is smooth and fine. In addition, we tested the solubility of chitosan (a) and TMC (b) in aqueous solution. When the concentration is the same (5 mg/ml), chitosan solution is obviously turbid and insoluble while TMC solution showed a clear and transparent state. The overlaid FTIR spectra of chitosan and TMC were displayed in Figure.S1B, the main proof of trimethylation of chitosan is verified by an absorption band at 1474 cm^{-1} present on the spectrum of TMC assigned to an asymmetric stretching of C–H bonds of methylated chitosan quaternary salts, which is not observed on the spectrum of chitosan. Another proof of chitosan modification is the absence of the band centered at 1592 cm^{-1}in the spectrum of TMC. Which was related to the angular deformation of N–H bonds of the amino group on chitosan. Furthermore, the typical bands in the scope of 2800~3500 cm^{-1} emerge at higher wavenumber on TMC spectrum while they are less intense than those on the chitosan spectrum. The absorption band at 3354 cm^{-1}, 2870 cm^{-1} and 1024 cm^{-1} on chitosan spectrum and 3348 cm^{-1}, 2943 cm^{-1}, 1037 cm^{-1} on TMC spectrum were assigned to the O–H bond, C–H bond and C–O bond respectively. The intensity of the absorption band at 1649 cm^{-1} on the TMC spectrum assigned to the axial stretching of C=O bond of the acetamide group may be because of the intermolecular hydrogen bond [1]. The XRD diffractograms of chitosan and TMC are demonstrated in Figure.S1C. The XRD spectrum of chitosan shows one main peak at 2θ=19.78°, which verifies the existence of crystalline domains on chitosan. Obvious differences can be found between XRD profiles of chitosan and TMC, there are two peaks at 2θ=31.40° and 2θ=45.15° on the XRD spectrum of TMC. In a word, the above results show that we successfully modified chitosan into TMC.

We further investigated the stability of Cel/PT-LbL Lipo when it was stored at 4 °C for one month. The particle size, zeta potential, PDI value and TEM image of the fresh Cel/PT-LbL Lipo sample and the sample with 1 month storage at 4 °C were determined, respectively. As shown in Table S1 and Figure.S2, Cel/PT-LbL Lipo had good storage stability.

Table S1. Particle size, zeta potential, and PDI of Cel/PT-LbL Lipo at day 0 and day 30 after storage at 4 °C for 1 month.

Cel/PT-LbL Lipo	Particle Size (nm)	Zeta Potential (mV)	PDI Value (%)
Day 0	179.95 ± 4.31	−10.0 ± 0.15	23.33 ± 0.59
Day 30	175.77 ± 1.59	−8.57 ± 0.15	22.00 ± 2.50

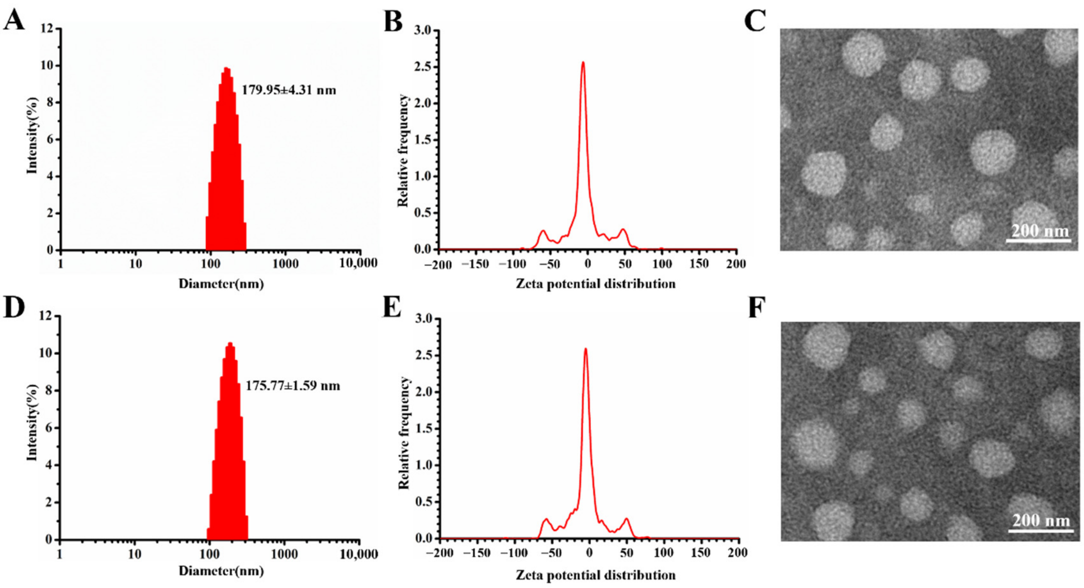

Figure S2. Comparison of particle size, potential and TEM morphology of Cel/PT-LbL Lipo at day 0 (**A**, **B**, **C**) and day 30 (**D**, **E**, **F**) after storage at 4 °C for 1 month.

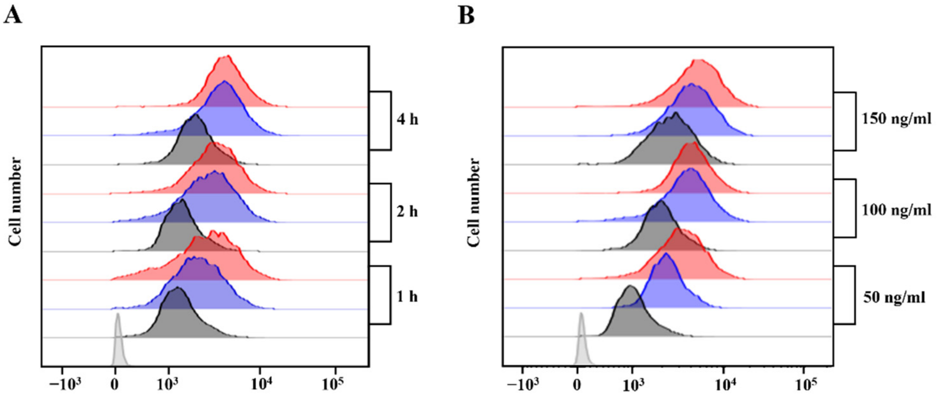

Figure S3. Cellular uptake advantages of C_6/TMC Lipo on colonic epithelial NCM460 cells. (**A**) Quantitative determination of the time-dependent cellular uptake by FCM. (**B**) Quantitative determination of the dose-dependent cellular uptake by FCM.

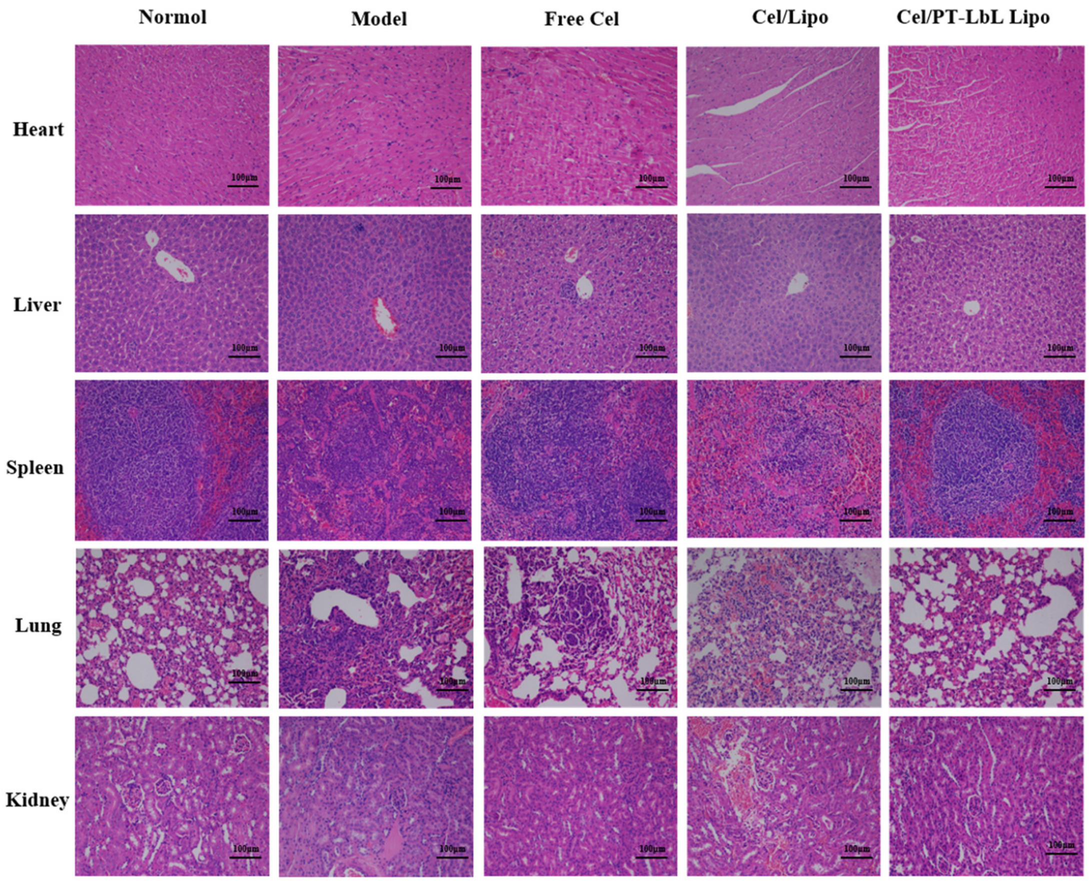

Figure S4. The representative H&E staining of heart, liver, spleen, lung, and kidney tissues. Scale bar is 100 μm.

The sections of the major organs from all mouse groups after treatment shown in Figure.S4, displayed no obvious histopathological damage in the Cel/PT-LbL Lipo groups. After treatment with Free Cel or Cel/ Lipo, H&E staining results showed certain cardiac, kidney, and lung toxicity. It can be seen from the figure that the arrangement of myocardial fibers was disordered and the gap widened and glomerulopathy in renal sections. On the other hand, compared with Cel/PT-LbL Lipo, Cel/ Lipo had more weight loss and higher mortality. This phenomenon could be attributed to the immune system suppression of liposomes which can engender depletion of phagocytic functions of mononuclear phagocytic system and suppress cytokine production from immune cells [10]. Therefore, the inflammation in mice was increased which led to increased mortality. However, when TMC and pectin were encapsulated in the surface of liposomes, their strong adhesion and colon targeting properties could significantly reduce the mortality and the toxicity of Free Cel.

References:

1. Pardeshi, C.V.; Belgamwar, V.S. Controlled synthesis of N,N,N-trimethyl chitosan for modulated bioadhesion and nasal membrane permeability. *Int. J. Biol. Macromol.* **2016**, *82*, 933–944.
2. Martins, A.F.; Bueno, P.V.A.; Follmann, H.D.M.; Nocchi, S.R.; Nakamura, C.V.; Rubira, A.F. and Muniz, E.C. Synthesis, characterization, and cytotoxicity of TMC-graft-poly (vinyl alcohol) copolymers. *Carbohydr. Res*. **2013**, *381*, 153–160.
3. Ribeiro, L.N.M.; Alcantara, A.C.S.; Darder, M.; Aranda, P.; Araujo-Moreira, F.M. and Ruiz-Hitzky, E. Pectin-coated chitosan-LDH bionanocomposite beads as potential systems for colon-targeted drug delivery. *Int. J. Pharm*. **2013**, 463, 1–9.
4. Monk, J.M.; Lepp, D.; Zhang, C.P.; Wu, W.; Zarepoor, L.; Lu, J.T.; Pauls, K.P.; Tsao, R.; Wood, G.A.; Robinson, L.E. and Power, K.A. Diets enriched with cranberry beans alter the microbiota and mitigate colitis severity and associated inflammation. *J. Nutr. Biochem*. **2016**, *28*, 129–139.
5. Shishir, M.R.I.; Karim, N.; Xie, J.; Rashwan, A.K. and Chen, W. Colonic delivery of pelargonidin-3-O-glucoside using pectin-chitosan-nanoliposome: Transport mechanism and bioactivity retention. *Int. J. Biol. Macromol*. **2020**, *159*, 341–355.

6. Yoo, C.Y.; Seong, J.S. and Park, S.N. Preparation of novel capsosome with liposomal core by layer-by-Layer self-assembly of sodium hyaluronate and chitosan. *Colloids Surf., B*. **2016**, *144*, 99–107.
7. Luo, R.; Lin, M.; Zhang, C.; Shi, J.; Zhang, S.; Chen, Q.; Hu, Y.; Zhang, M.; Zhang, J. and Gao, F. Genipin-crosslinked human serum albumin coating using a tannic acid layer for enhanced oral administration of curcumin in the treatment of ulcerative colitis. *Food Chem*. **2020**, *330*, 127241.
8. Liu, H.; Yang, X.; Nie, S.; Wei, L.; Zhou, L.; Liu, H.; Tang, R. and Pan, W. Chitosan-based controlled porosity osmotic pump for colon-specific delivery system: Screening of formulation variables and in vitro investigation. *Int. J. Pharm*. **2007**, *332*, 115–124.
9. Gupta, R.A.; Motiwala, M.N.; Dumore, N.G.; Danao, K.R. and Ganjare, A.B. Effect of piperine on inhibition of FFA induced TLR4 mediated inflammation and amelioration of acetic acid induced ulcerative colitis in mice. *J. Ethnopharmacol*. **2015**, *164*, 239–246.
10. Zahednezhad, F.; Saadat, M.; Valizadeh, H.; Zakeri-Milani, P. and Baradaran, B. Liposome and immune system interplay: Challenges and potentials. *J. Controlled Release*. **2019**, *305*, 194–209.
